# Mutant *B3GALT6* in a Multiplex Family: A Dominant Variant Co-Segregated With Moderate Malformations

**DOI:** 10.3389/fgene.2022.824445

**Published:** 2022-06-06

**Authors:** Fang Shen, Yongjia Yang, Yu Zheng, Ming Tu, Liu Zhao, Zhenqing Luo, Yuyan Fu, Yimin Zhu

**Affiliations:** ^1^ The Laboratory of Genetics and Metabolism, Pediatrics Research Institute of Hunan Province, Hunan Children’s Hospital, Hengyang Medical School, University of South China, Changsha, China; ^2^ Emergency Research Institute of Hunan Province, Hunan People’s Hospital, Changsha, China

**Keywords:** *B3GALT6*, autosomal dominant, heterozygous variant, dominant-negative effect, EDS

## Abstract

*B3GALT6* is a well-documented disease-related gene. Several *B3GALT6*-recessive variants have been reported to cause Ehlers–Danlos syndrome (EDS). To the best of our knowledge, no dominant *B3GALT6* variant that causes human disease has been reported. In 2012, we reported on a three-generation, autosomal-dominant family with multiple members who suffered from radioulnar joint rotation limitation, scoliosis, thick vermilion of both lips, and others, but the genetic cause was unknown. Here, exome sequencing of the family identified mutant *B3GALT6* as the cause of the multiplex affected family. We observed that, in the compound heterozygous pattern (i.e., c.883C>T:p.R295C and c.510_517del:p.L170fs*268), mutant *B3GALT6* led to severe consequences, and in the dominant pattern, an elongated *B3GALT6* variant co-segregated with moderate phenotypes. The functional experiments were performed *in vitro*. The R295C variant led to subcellular mislocalization, whereas the L170fs*268 showed normal subcellular localization, but it led to an elongated protein. Given that most of the catalytic galactosyltransferase domain was disrupted for the L170fs*268 (it is unlikely that such a protein has activity), we propose that the L170fs*268 occupies the normal *B3GALT6* protein position in the Golgi and exerts a dominant-negative effect.

## Introduction

Glycosaminoglycan (GAG), a key component of the extracellular matrix, is essential for the development and maintenance of bone, cartilage, skin, and other tissues. GAG synthesis is initiated by the formation of a tetrasaccharide linker region attached to a serine residue in the proteoglycan core protein (Prydz and Dalen, 2000). Synthesis of the linker region involves four successive steps catalyzed by distinctive enzymes: xylosyltransferases I/II (encoded by *XYLT1* and *XYLT2*) (Götting et al., 2000; Bui et al., 2014; Munns et al., 2015; Umair et al., 2018; LaCroix et al., 2019), galactosyltransferase I (*β*4GalT7, encoded by *B4GALT7*) (Guo et al., 2013; Cartault et al., 2015; Ritelli et al., 2017; Sandler-Wilson et al., 2019), galactosyltransferase II (*β*3GalT6, encoded by *B3GALT6*) (Malfait et al., 2013; Nakajima et al., 2013), and glucuronosyltransferase I (GlcAT-I, encoded by *B3GAT3*) (Yauy et al., 2018; Colman et al., 2019). Recessive pathogenic variants of any of these four genes lead to severe skeletal deformities and connective tissue disruptions.

*B3GALT6* (NM_080605.3) is a single-exon gene on chromosome 1p36.33, which encodes beta-1,3-galactosyltransferase 6 (*β*3GalT6). The enzyme localizes predominantly in the Golgi apparatus and catalyzes the addition of a third galactose to the second galactose of the GAG linker region. In 2013, Malfait et al. (2013) and Nakajima et al. (2013) reported *B3GALT6* pathogenic variants in human diseases. Since then, dozens of *B3GALT6* variants in the recessive status have been identified in approximately 40 unrelated families with Ehlers–Danlos syndrome (EDS) (Sellars et al., 2014; Vorster et al., 2015; Alazami et al., 2016; Trejo et al., 2017; Ben-Mahmoud et al., 2018; Van Damme et al., 2018; Caraffi et al., 2019).

In 2012, we reported on an autosomal-dominant (AD), three-generation family with multiple members presenting with radioulnar limitation, scoliosis, thick vermilion of both lips, and a shortened and thickened femur neck (Zhu et al., 2012). Exome sequencing (ES) of the family was performed. Variant analysis and validation tests identified the following: 1) *B3GALT6*-recessive (compound heterozygous) variants led to severe phenotypes (EDS); 2) the frameshift-elongated variant (c.510_517del:p.L170fs*268) segregated with moderate deformities in three members of the family in a dominant manner. Functional experiments confirmed that the R295C variant was loss-of-function, but the elongated variant (p.L170fs*268) may exert a dominant-negative effect. This is the first report on *B3GALT6*-dominant variant leading to disease.

## Materials and Methods

### Study Subjects

The study was approved by the Academic Committee of Hunan Children’s Hospital (approval number: HCHLL58, Changsha City, Hunan Province, China). All family members provided written informed consent to participate in this study.

### Exome Sequencing

**FIGURE 1:**
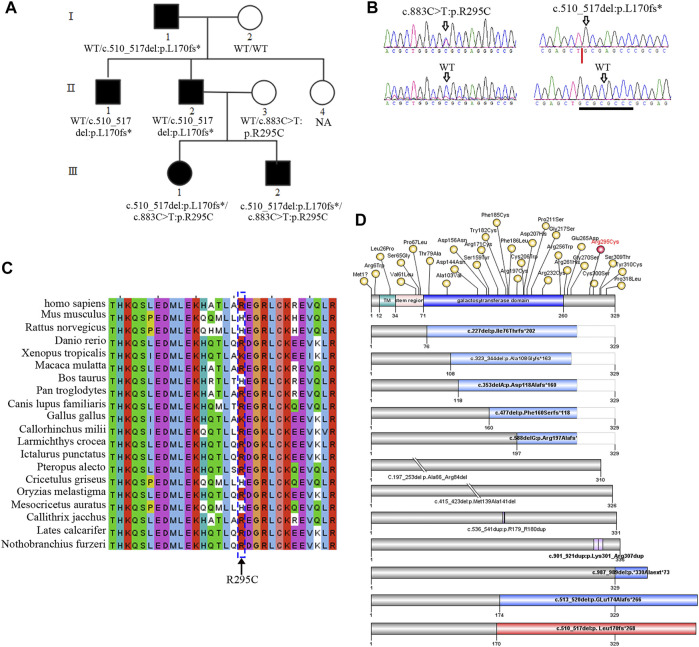
*B3GALT6* mutated in an autosomal-dominant family. **(A)** Pedigree with five affected members. NA, genomic DNA was unavailable. **(B)** Two variants in the family, i.e., c.883C>T:p.R295C and c.510_517del:p. L170fs*268. Note: the trace figure of c.510_517del was identified by Sanger sequencing after the PCR fragment TA-clone experiment. **(C)** Protein sequence alignment of the residue 295 (arginine) across different species. **(D)** Schematic view of *β*3Galt6 protein and the position of the pathogenic variants reported so far. The upper bar represents the domain structure of wild-type (WT) *β*3Galt6. Missense variants identified so far were depicted above the WT-*β*3Galt6, and indel variants were depicted below the WT-*β*3Galt6. TM, transmembrane region. Note: variants depicted as red were found in this study.

ES was carried out on seven individuals (I:1, I:2, II:1, II:2, II:3, III:1, and III:2) (Figure 1A). The detailed ES pipelines have been reported previously (Yang et al., 2019). Briefly, genomic DNA was fragmented into 180–280 bp segments, libraries were prepared and captured using an Agilent SureSelect Human All Exon V6 kit for each individual, and the effective concentration of each sample was subsequently sequenced on an Illumina HiSeq X Ten Sequencing system (Illumina Inc., San Diego, CA, United States). The raw BCL file was then converted into a raw FASTQ file. Raw reads were filtered using FastQC to remove low-quality reads. Clean reads were then mapped to the reference genome GRCh37 using Bwa. After removing duplications, SNV and InDel were called and annotated using GATK. For each sample, 11.9 G bases were obtained. The average yield was 16.6 Gb with an error rate of <0.1%. Furthermore, >90% bases had a Phred quality score of ≥30 (Q30).

### Cell Transfection and Western Blot

The empty vector pCMV-14-3 ×flag or *B3GALT6* expression constructs (WT, R295C, or L170fs*268) were separately transfected into HeLa cells using Lipofectamine 3000 (Invitrogen, L3000-015) for 48 h. Protein extracts were collected and separated by 10% SDS-PAGE, electrotransferred to a polyvinylidene fluoride (PVDF) membrane (0.45 µm, Merck Millipore Ltd.), blocked with buffer containing 5% non-fat milk, and incubated with mouse monoclonal anti-flag antibody (1:2500) (Sigma, F1804) overnight at 4°C and HRP-conjugated secondary antibodies for 1 h at room temperature and developed with an enhanced chemiluminescence HRP substrate kit (Millipore, WBKLS0500). The membrane was visualized using an iBright FL1500 imaging system (Invitrogen).

### Cellular Immunofluorescence

HeLa cells were seeded on coverslips in 24-well culture plates. When the cells reached 80% confluence, the *B3GALT6* expression constructs were transfected for 48 h. Cells were blocked with 4% paraformaldehyde for 30 min, permeabilized with 0.2% Triton X-100 for 10 min, and blocked with 5% BSA for 1 h at room temperature. The solution was discarded, and the mouse monoclonal anti-flag antibody (1:500) (Sigma, F1804) and rabbit anti-GOLPH4 antibody (1:400) (Abcam, ab28049) were added onto the coverslips overnight at 4°C in a moist environmental box. The secondary antibody Cy3-conjugated goat anti-mouse IgG (Origene, TA130012) and FITC-conjugated goat anti-rabbit IgG (Origene, TA130021) were incubated for 1 h at room temperature in a dark place, and then DAPI was added onto coverslips for 5 min. Coverslips were mounted on the slide. Cell images were captured with laser scanning confocal microscopy LSM 800.

## Results

### *B3GALT6* Variants in the Family

As previously reported, an autosomal-dominant family was investigated (Figure 1A; Table 1), and we suspected that the family was affected by Giuffre–Tsukahara syndrome at that time (Zhu et al., 2012). ES was successfully performed on seven family members (Figure 1A). After obtaining the variant list, we first focused on the loss-of-function variants (including canonical splicing variants, indels, or other truncating variants) as well as elongation frameshift variants. Because AD inheritance was observed in the index family (Figure 1A), and the disease is extremely rare, we focused on heterozygous, rare, coding variants with AF less than 0.0001 (Exac_eas; gnomAD_eas; 1000G_eas; gomAD_genome all). Then, we only considered the variants (absent in our 700 in-house ES data) that co-segregated with the affected status in the index family; one variant remained, c.510_517del:p. L170fs*268, at *B3GALT6*. Because *B3GALT6* is a known gene for the connective tissue disorder (EDS), we reanalyzed all *B3GALT6* rare variants with AF less than 0.001 (including missense variants) and identified another *B3GALT6* missense variant, i.e., c.883C>T:p.R295C on three family members (Ⅱ:3, Ⅲ:1, and Ⅲ:2). Sanger sequencing validation confirmed that two family members with severe phenotypes (Ⅲ:1 and Ⅲ:2) had *B3GALT6* compound heterozygous variants (c.883C>T:p.R295C/c.510_517del:p. L170fs*268) (Figures 1A,B; Table 1). Unaffected family member Ⅱ:3 was heterozygous for c.883C>T:p.R295C variant (Figures 1A,B; Table 1), and family members Ⅰ:1, Ⅱ:1, and Ⅱ:2 with moderate phenotypes suffered by c.510_517del:p.L170fs*268 heterozygous variant (Figures 1A,B; Table 1).

**TABLE 1:** Manifestations and radiographic findings of the individuals in the family.

Family ID	I:1	II:1	II:2	III:1	III:2
General information					
Gender	Male	Male	Male	Female	Male
Age (years)	67	41	38	14	7
Birth weight (g)	ND	ND	ND	3,030	2,850
Intelligence	Normal	Normal	Normal	Mild deficit in speech	Normal
Height (cm)	169	177	175	139 (158.6)	109 (122)
Weight (kg)	70	76	67	35 (50.5)	18.1 (22.9)
Craniofacial					
Thick vermilion lips	+	+	+	+	+
Flat malar region	+	+	−	+	+
High forehead	−	−	−	+	+
Epicanthal folds	−	−	−	+	+
Prominent eyes	−	−	−	+	+
Blue sclerae	−	−	−	+	+
Protruding ear	−	−	−	+	+
Musculoskeletal					
Scoliosis	+	+	+	++	++
Fifth-finger clinodactyly	+	-	-	+	+
Restricted elbow movement	+	+	+	+	+
Joint hypermobility	−	−	−	++	++
Radiological features					
Radioulnar synostosis	+	−	−	+	+
Shortened and thickened femoral neck	ND	ND	ND	+	+
Others	Aclasis of right humerus and ulna	—	—	Soft, doughy skin	Barrel chest and soft, doughy skin

ND, no data.

### Functional Characteristics of *β*3Galt6 Variants

The c.883C>T:p.R295C is not located in the catalytic galactosyltransferase domain (Figure 1D). Protein sequence alignment indicated that residue 295 (arginine) was not highly conserved across different species (Figure 1C). Meanwhile, in the gnomAD database, there was a very low frequency (AF = 0.0005) in the East Asian population, and 13/23 silico software predicted it to be benign or tolerant (Supplementary Table S1). The c.510_517del:p.L170fs*268 variant was not found in the ExAC, 1000G, and gnomAD databases, and it was not observed in 700 exome databases of our in-house datasheet that are unrelated to skeletal diseases (data not shown).

According to the first in-frame ATG in the reference sequence (NM_080605.4), the c.510_517del variant led to a frameshift mutation and introduced a termination codon delay. Consequently, it was predicted that the mutant-elongated protein contained 438 amino acids in contrast to the wild-type protein, which contained 329 amino acids (Figure 1; Supplementary Material S1).

**FIGURE 2:**
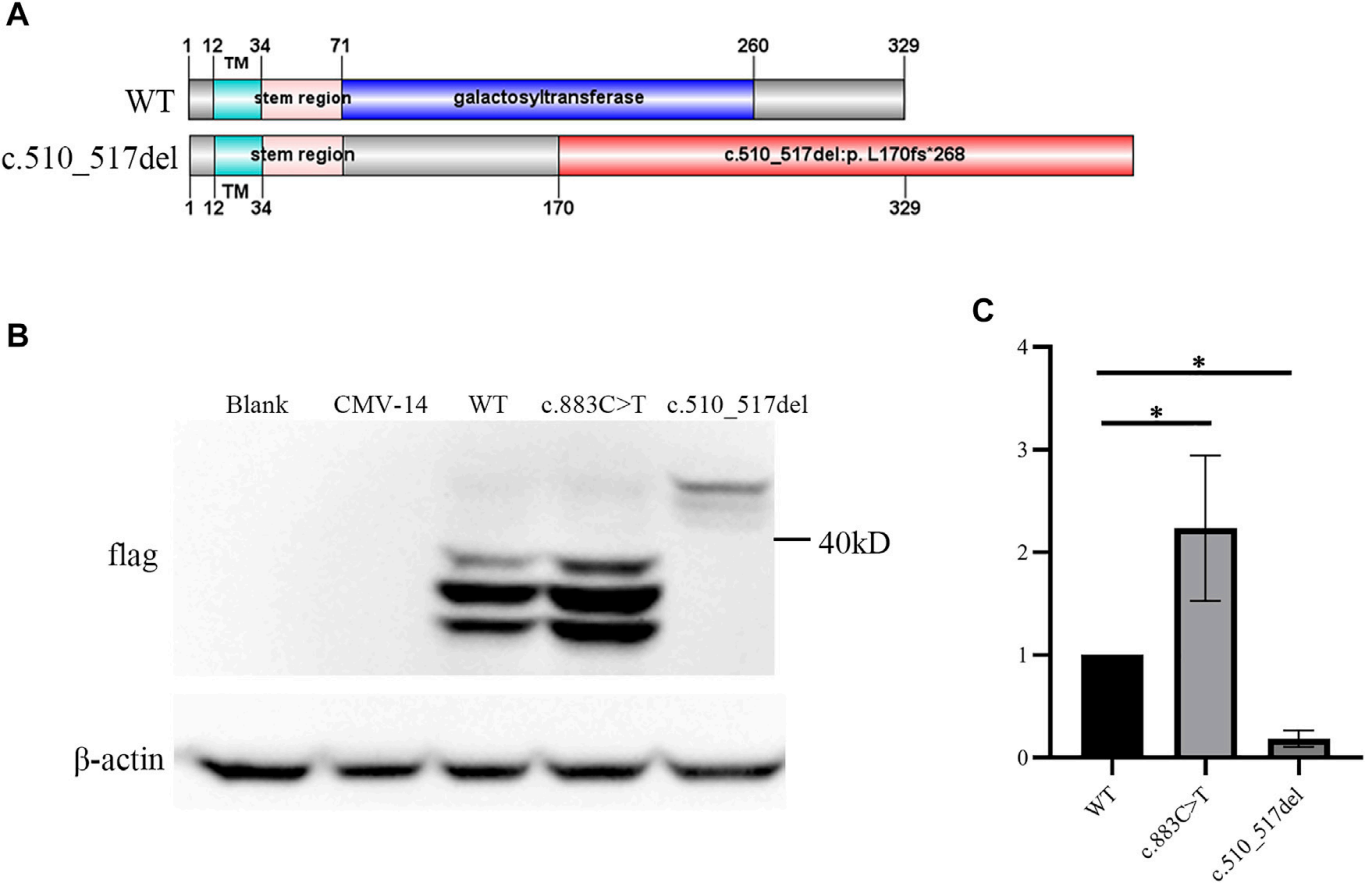
c.510_517del:p. L170fs*268 linked an elongated product. **(A)** Upper panel represents the wild-type (WT) *β*3Galt6. Lower panel represents the c.510_517del:p. L170fs*268 elongation variant. Note: in wild-type *β*3Galt6, the catalytic galactosyltransferase domain was depicted in blue; red: frameshift amino acid sequence caused by the deletion. **(B)** Western blot analysis of lysates from HeLa cells expressing WT and mutant *B3GALT6.* CMV-14, empty plasmid. **(C)** Relative density of *β*3Galt6. *n* = 4 per group, *p < 0.05.

*β*3Galt6 mainly functions in the Golgi (Bai et al., 2001). Previous studies have identified *B3GALT6* variants exerting pathogenic effects by subcellular mislocalization or unstable structure of the mutant protein or by the unstable/incomplete transcript at the RNA level (Nakajima et al., 2013; Ben-Mahmoud et al., 2018). We then overexpressed *β*3Galt6 (WT, R295C, or L170fs*268) in the HeLa cells. Western blot analysis showed that: 1) the size of the R295C protein was the same as that of the wild-type protein, but the expression of R295C was increased significantly (Figures 2B,C); 2) in contrast to wild-type *β*3Galt6, a band with a larger molecular weight was observed for the frameshift mutant protein (L170fs*268), and a significantly reduced expression was observed (Figures 2B,C).

**FIGURE 3:**
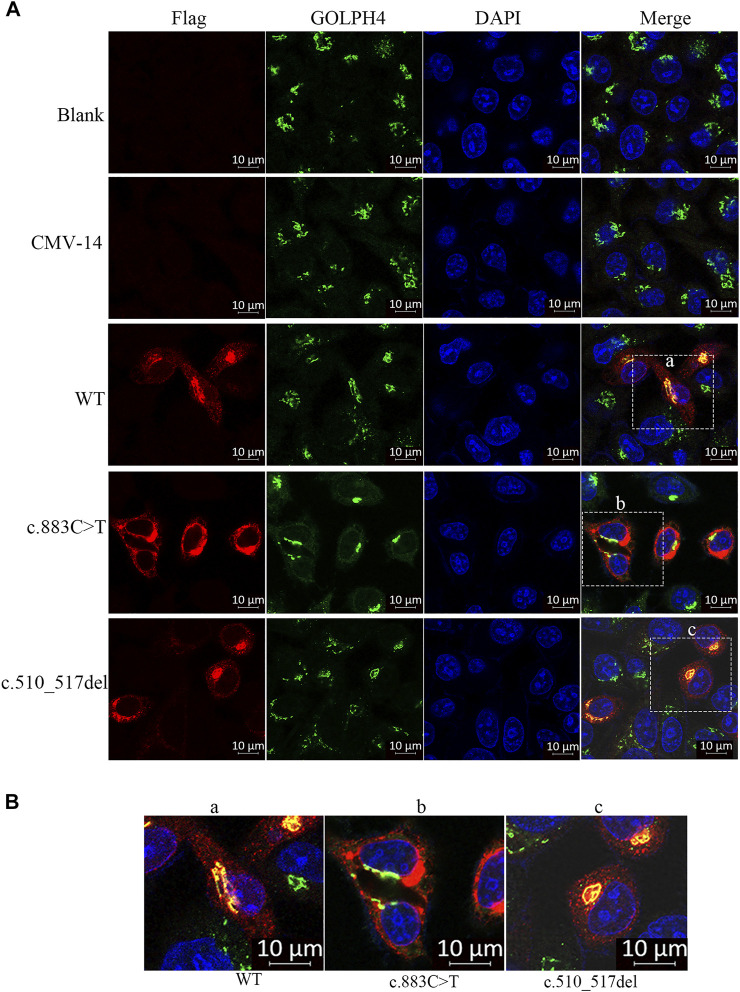
Subcellular localization of *β*3Galt6. **(A)** HeLa cells were transfected with plasmid carrying the wild-type (WT), the c.883C>T, or the c.510_517del variant. Cells were stained with anti-FLAG (red), anti-GOLPH4 (green), and DAPI (blue). **(B)** Enlarged images of **(a–c)** point of **(A)**.

Second, we checked the subcellular localization of *β*3Galt6 (WT, R295C, or L170fs*268). In contrast to WT-*β*3Galt6 expressed in the Golgi (as it co-localized with GOLPH4, which is a marker of the Golgi) (Figure 3), the mutant R295C protein was found in the cytoplasm but not in the Golgi (Figure 3), indicating that the mutant R295C protein was functionally null. In contrast, *β*3Galt6-L170fs*268 was located in the Golgi compartment, as it overlaps with GOLPH4. According to the ACMG/AMP criteria (Richards et al., 2015), both the variants were classified as pathogenic; the evaluation results of the L170fs*268 variant were PVS1, PM1, PM2, PP1, and PP4; and for the R295C variant, the evaluation results were PS3, PM2, PM3, PP1, and PP4.

### Heterozygous *B3GALT6*- L170fs*268 Leading to a Moderate Phenotype

The family members Ⅲ:1 and Ⅲ:2 exhibited severe phenotypes (which were consistent with EDS). Other individuals in the family (Ⅰ:1, Ⅱ:1, and Ⅱ:2) did not meet the criteria for EDS and clearly exhibited moderate phenotypes (Table 1 and ref 22). The *in vitro* study identified that a plasmid with c.510_517del:p. L170fs*26 variant can express an elongated protein (Figure 2), and the mutant elongated protein can be correctly localized in the Golgi. Given that the L170fs*26 frameshift variant resulted in about half of the catalytic galactosyltransferase domain being disrupted (Figure 2A), we propose that the elongated protein (having no catalytic function) occupies the Golgi position disrupting normal GAG production, resulting in a dominant-negative effect.

In brief, the present study identified two novel B3GALT6 pathogenic variants in a multiplex family: 1) in the recessive status, mutant B3GALT6 causing EDS; and 2) in the dominant status, the elongation-mutant *β*3Galt6 causing moderate phenotypes (although not reaching the level of a typical EDS).

## Discussion

As a key enzyme in GAG synthesis, *β*3Galt6 is encoded by the *B3GALT6* gene (Bai et al., 2001). In an autosomal-recessive manner, pathogenic variants of *B3GALT6* lead to a multisystem disorder mainly comprising bone deformity and connective tissue disruption, that is, spondylodysplastic EDS (Malfait et al., 2013; Nakajima et al., 2013; Van Damme et al., 2018; Caraffi et al., 2019). Approximately, 60 patients from 40 families have been reported to have mutated *B3GALT6* (Malfait et al., 2013; Nakajima et al., 2013; Sellars et al., 2014; Ritelli et al., 2015; Vorster et al., 2015; Alazami et al., 2016; Trejo et al., 2017; Ben-Mahmoud et al., 2018; Van Damme et al., 2018; Caraffi et al., 2019; Zhang et al., 2020; Descartes et al., 2021; Leoni et al., 2021). Among these patients, 41 *B3GALT6* pathogenic variants have been reported (Figure 1C), including two start-codon loss (Nakajima et al., 2013; Van Damme et al., 2018), two in-frame deletion variants (Nakajima et al., 2013; Van Damme et al., 2018), two in-frame duplication variants (Alazami et al., 2016; Trejo et al., 2017), five frameshift variants that lead to truncation of the protein (Malfait et al., 2013; Nakajima et al., 2013; Ritelli et al., 2015; Van Damme et al., 2018; Caraffi et al., 2019), two frameshift variants that lead to elongation of the protein (Van Damme et al., 2018; Caraffi et al., 2019), and 28 missense variants (Malfait et al., 2013; Nakajima et al., 2013; Sellars et al., 2014; Ritelli et al., 2015; Vorster et al., 2015; Alazami et al., 2016; Trejo et al., 2017; Ben-Mahmoud et al., 2018; Van Damme et al., 2018; Caraffi et al., 2019; Zhang et al., 2020; Descartes et al., 2021; Leoni et al., 2021). Frameshift variants that lead to protein elongation (combined with other missense variants) have been reported in several severe sporadic patients (Van Damme et al., 2018; Caraffi et al., 2019).

Given that 41 B3GALT6 pathogenic variants (including elongation variants) have been reported on EDS as described previously, none of these variants caused a dominant phenotype. In the present study, we identified two novel *B3GALT6* pathogenic variants, i.e., the c.883C>T:p.R295C and the c.510_517del:p.L170fs*268. The *B3GALT6*-prolonged variant (c.510_517del:p.L170fs*268) was detected in five family members. In two members (who combined with another point R295C variant), severe phenotypes (typical components of EDS) were observed, which is consistent with previous studies, that is, in the recessive status, B3GALT6 variants lead to EDS. The novelty of the present study is that in the other three family patients (Ⅰ:1, Ⅱ:1, and Ⅱ:2), the heterozygous L170fs*268 variant co-segregated with less severe but obvious phenotypes, such as radioulnar joint limitation, “S”-shaped scoliosis, and thick vermilion of the lips.

To test the functional consequences of the elongation variant, we expressed *B3GALT6* L170fs*268 protein in HeLa cells. Western blot analysis identified an apparently elongated band (with a significantly reduced amount), which was consistent with the cell immunofluorescence assay in which the *B3GALT6* L170fs*268 protein could be detected in the cytoplasm. However, in the immunofluorescence assay, we did not observe a reduction in the L170fs*268 protein. Such a difference in protein amount between the Western blot assay and the immunofluorescence assay may contribute to the L170fs*268 proteins having less stability when they are separated from the *in vivo* cells.

Similar to WT-*β*3Galt6, which is expressed in the Golgi, the *β*3Galt6-L170fs*268 is also located in the Golgi. Therefore, the previous pathomechanism (subcellular mislocalization or haploinsufficiency) cannot explain the heterozygous *β*3Galt6-L170fs*268 variant-causing phenotypes. Given that the heterozygous *β*3Galt6-L170fs*268 variant co-segregated with moderate phenotypes in the family produced an elongated protein, and the mutant protein could localize to the Golgi apparatus, we propose that the disease-causing mechanism for this elongation variant is the L170fs*268 that occupies the Golgi apparatus and disrupts the normal CAG production. Nevertheless, to confirm the novel *β*3Galt6-pathogenic mechanism, further studies, such as galactosyltransferase activity assay, GAG synthesis assay, and CS and HS chain quantifications, are warranted to elucidate the detailed mechanism.

In conclusion, we identified heterozygous *B3GALT6*-causing phenotypes that implicate a new dominant inheritance pattern of the mutated *B3GALT6* or CAG syntheses.

## Data Availability

The datasets presented in this study can be found in online repositories. The names of the repository/repositories and accession number(s) can be found in the article/Supplementary Material.

## References

[B1] Alazami A. M. Al-Qattan S. M. Faqeih E. Alhashem A. Alshammari M. Alzahrani F. (2016). Expanding the Clinical and Genetic Heterogeneity of Hereditary Disorders of Connective Tissue. Hum. Genet. 135 (5), 525–540. 10.1007/s00439-016-1660-z 27023906

[B2] Bai X. Zhou D. Brown J. R. Crawford B. E. Hennet T. Esko J. D. (2001). Biosynthesis of the Linkage Region of Glycosaminoglycans. J. Biol. Chem. 276 (51), 48189–48195. 10.1074/jbc.M107339200 11551958

[B3] Ben-Mahmoud A. Ben-Salem S. Al-Sorkhy M. John A. Ali B. R. Al-Gazali L. (2018). A B3GALT6 Variant in Patient Originally Described as Al-Gazali Syndrome and Implicating the Endoplasmic Reticulum Quality Control in the Mechanism of Some β3GalT6-pathy Mutations. Clin. Genet. 93 (6), 1148–1158. 10.1111/cge.13236 29443383

[B4] Bui C. Huber C. Tuysuz B. Alanay Y. Bole-Feysot C. Leroy J. G. (2014). Xylt1 Mutations in Desbuquois Dysplasia Type 2. Am. J. Hum. Genet. 94 (3), 405–414. 10.1016/j.ajhg.2014.01.020 24581741 PMC3951945

[B5] Caraffi S. G. Maini I. Ivanovski I. Pollazzon M. Giangiobbe S. Valli M. (2019). Severe Peripheral Joint Laxity Is a Distinctive Clinical Feature of Spondylodysplastic-Ehlers-Danlos Syndrome (EDS)-B4GALT7 and Spondylodysplastic-EDS-B3galt6. Genes 10, 799. 10.3390/genes10100799 PMC6826576 31614862

[B6] Cartault F. Munier P. Jacquemont M.-L. Vellayoudom J. Doray B. Payet C. (2015). Expanding the Clinical Spectrum of B4galt7 Deficiency: Homozygous P.R270c Mutation with Founder Effect Causes Larsen of Reunion Island Syndrome. Eur. J. Hum. Genet. 23 (1), 49–53. 10.1038/ejhg.2014.60 24755949 PMC4266744

[B7] Colman M. Van Damme T. Steichen-Gersdorf E. Laccone F. Nampoothiri S. Syx D. (2019). The Clinical and Mutational Spectrum of B3gat3 Linkeropathy: Two Case Reports and Literature Review. Orphanet J. Rare Dis. 14 (1), 138. 10.1186/s13023-019-1110-9 31196143 PMC6567438

[B8] Descartes M. Melenevsky Y. V. Rudy N. Smith K. Callaway K. Parker J. S. (2021). Keratoconus in a Patient with B3GALT6 ‐related Disorder. Clin. Genet. 99 (6), 849–850. 10.1111/cge.13940 33631843

[B9] Götting C. Kuhn J. Zahn R. Brinkmann T. Kleesiek K. (2000). Molecular Cloning and Expression of Human UDP-D-Xylose:Proteoglycan Core Protein β-d-Xylosyltransferase and its First Isoform XT-II. J. Mol. Biol. 304 (4), 517–528. 10.1006/jmbi.2000.4261 11099377

[B10] Guo M. H. Stoler J. Lui J. Nilsson O. Bianchi D. W. Hirschhorn J. N. (2013). Redefining the Progeroid Form of Ehlers-Danlos Syndrome: Report of the Fourth Patient withB4GALT7deficiency and Review of the Literature. Am. J. Med. Genet. 161A (10), a–n. 10.1002/ajmg.a.36128 PMC3788078 23956117

[B11] LaCroix A. J. Stabley D. Sahraoui R. Adam M. P. Mehaffey M. Kernan K. (2019). Ggc Repeat Expansion and Exon 1 Methylation of Xylt1 Is a Common Pathogenic Variant in Baratela-Scott Syndrome. Am. J. Hum. Genet. 104 (1), 35–44. 10.1016/j.ajhg.2018.11.005 30554721 PMC6323552

[B12] Leoni C. Tedesco M. Radio F. C. Chillemi G. Leone A. Bruselles A. (2021). Broadening the Phenotypic Spectrum of Beta3GalT6 ‐associated Phenotypes. Am. J. Med. Genet. 185 (10), 3153–3160. 10.1002/ajmg.a.62399 34159694

[B13] Malfait F. Kariminejad A. Van Damme T. Gauche C. Syx D. Merhi-Soussi F. (2013). Defective Initiation of Glycosaminoglycan Synthesis Due to B3galt6 Mutations Causes a Pleiotropic Ehlers-danlos-syndrome-like Connective Tissue Disorder. Am. J. Hum. Genet. 92 (6), 935–945. 10.1016/j.ajhg.2013.04.016 23664118 PMC3675258

[B14] Munns C. F. Fahiminiya S. Poudel N. Munteanu M. C. Majewski J. Sillence D. O. (2015). Homozygosity for Frameshift Mutations in Xylt2 Result in a Spondylo-Ocular Syndrome with Bone Fragility, Cataracts, and Hearing Defects. Am. J. Hum. Genet. 96 (6), 971–978. 10.1016/j.ajhg.2015.04.017 26027496 PMC4457947

[B15] Nakajima M. Mizumoto S. Miyake N. Kogawa R. Iida A. Ito H. (2013). Mutations in B3galt6, Which Encodes a Glycosaminoglycan Linker Region Enzyme, Cause a Spectrum of Skeletal and Connective Tissue Disorders. Am. J. Hum. Genet. 92 (6), 927–934. 10.1016/j.ajhg.2013.04.003 23664117 PMC3675233

[B16] Prydz K. Dalen K. T. (2000). Synthesis and Sorting of Proteoglycans. J. Cell. Sci. 113 (Pt 2), 193–205. 10.1242/jcs.113.2.193 10633071

[B17] Richards S. Aziz N. Bale S. Bick D. Das S. Gastier-Foster J. (2015). Standards and Guidelines for the Interpretation of Sequence Variants: a Joint Consensus Recommendation of the American College of Medical Genetics and Genomics and the Association for Molecular Pathology. Genet. Med. 17 (5), 405–424. 10.1038/gim.2015.30 25741868 PMC4544753

[B18] Ritelli M. Chiarelli N. Zoppi N. Dordoni C. Quinzani S. Traversa M. (2015). Insights in the Etiopathology of Galactosyltransferase Ii (Galt-Ii) Deficiency from Transcriptome-wide Expression Profiling of Skin Fibroblasts of Two Sisters with Compound Heterozygosity for Two Novel B3galt6 Mutations. Mol. Genet. Metabolism Rep. 2, 1–15. 10.1016/j.ymgmr.2014.11.005 PMC5471164 28649518

[B19] Ritelli M. Dordoni C. Cinquina V. Venturini M. Calzavara-Pinton P. Colombi M. (2017). Expanding the Clinical and Mutational Spectrum of B4galt7-Spondylodysplastic Ehlers-Danlos Syndrome. Orphanet J. Rare Dis. 12 (1), 153. 10.1186/s13023-017-0704-3 28882145 PMC5590203

[B20] Sandler-Wilson C. Wambach J. A. Marshall B. A. Wegner D. J. McAlister W. Cole F. S. (2019). Phenotype and Response to Growth Hormone Therapy in Siblings with B4galt7 Deficiency. Bone 124, 14–21. 10.1016/j.bone.2019.03.029 30914273 PMC6551519

[B21] Sellars E. A. Bosanko K. A. Lepard T. Garnica A. Schaefer G. B. (2014). A Newborn with Complex Skeletal Abnormalities, Joint Contractures, and Bilateral Corneal Clouding with Sclerocornea. Seminars Pediatr. Neurology 21 (2), 84–87. 10.1016/j.spen.2014.04.007 25149931

[B22] Trejo P. Rauch F. Glorieux F. H. Ouellet J. Benaroch T. Campeau P. M. (2017). Spondyloepimetaphysial Dysplasia with Joint Laxity in Three Siblings with B3GALT6 Mutations. Mol. Syndromol. 8 (6), 303–307. 10.1159/000479672 29230159 PMC5701276

[B23] Umair M. Eckstein G. Rudolph G. Strom T. Graf E. Hendig D. (2018). Homozygous Xylt2 Variants as a Cause of Spondyloocular Syndrome. Clin. Genet. 93 (4), 913–918. 10.1111/cge.13179 29136277

[B24] Van Damme T. Pang X. Guillemyn B. Gulberti S. Syx D. De Rycke R. (2018). Biallelic B3galt6 Mutations Cause Spondylodysplastic Ehlers-Danlos Syndrome. Hum. Mol. Genet. 27 (20), 3475–3487. 10.1093/hmg/ddy234 29931299

[B25] Vorster A. A. Beighton P. Ramesar R. S. (2015). Spondyloepimetaphyseal Dysplasia with Joint Laxity (Beighton Type); Mutation Analysis in Eight Affected South African Families. Clin. Genet. 87 (5), 492–495. 10.1111/cge.12413 24766538

[B26] Yang Y. Zheng Y. Li W. Li L. Tu M. Zhao L. (2019). Smad6 Is Frequently Mutated in Nonsyndromic Radioulnar Synostosis. Genet. Med. 21 (11), 2577–2585. 10.1038/s41436-019-0552-8 31138930

[B27] Yauy K. Tran Mau-Them F. Willems M. Coubes C. Blanchet P. Herlin C. (2018). B3gat3-Related Disorder with Craniosynostosis and Bone Fragility Due to a Unique Mutation. Genet. Med. 20 (2), 269–274. 10.1038/gim.2017.109 28771243

[B28] Zhang J. Huang K. Dong G. (2020). Clinical and Genetic Analysis of a Child with Spondyloepimetaphyseal Dysplasia Type 1 and Joint Laxity. Zhonghua Yi Xue Yi Chuan Xue Za Zhi 37 (8), 887–890. 10.3760/cma.j.issn.1003-9406.2020.08.020 32761602

[B29] Zhu Y. Jin K. Mei H. Li L. Liu Z. Yang Y. (2012). A Family with Radio-Ulnar Synostosis, Scoliosis, and Thick Vermilion of Lips: A Novel Syndrome or Variant of Giuffrè-Tsukahara Syndrome? Am. J. Med. Genet. 158A (8), 2036–2042. 10.1002/ajmg.a.35478 22786695

