# Supplementary material for: Mutant B3GALT6 in a Multiplex Family: A Dominant Variant Co-Segregated With Moderate Malformations
Source: Front Genet. 2022 Jun 6;13:824445. doi: 10.3389/fgene.2022.824445 (PMC9207203; doi:10.3389/fgene.2022.824445)
Supplement: Supplementary file 1 [file DataSheet1.PDF]

The DNA sequence of wild-type *B3GALT6*

atgaagctgctgcggcgggcgtggcggcggcgggcggcgctaggcctgggcacgctggcgctgtgcggggcggcgctgctctacctggcg
cgctgcgcggccgagcccggggaccccagggcgatgtcgggccgcagcccgcctcccccgcgcccgcgcgcgccgccgccttcctggca
gtgctggtggccagcgcgccccgcgccgccgagcgccgcagcgtgatccgcagcacgtggcttgcgcggcgcggggccccgggcgacgtg
tgggcgcgctttgccgtgggcacggccggcctgggcgccgaggagcggcgcgccctggagcgggagcaggcgcggcacggggacctgct
gctgctgcccgcgctgcgcgacgcctacgaaaacctcacggccaaggtgctggccatgctggcctggctggacgagcacgtggccttcgag
ttcgtgctcaaggcggacgacgactccttcgcgcggctggacgcgctgctggccgagctgcgcgcccgcgagcccgcgcgccgccgccgcc
tctactggggcttcttctcgggccgcggccgcgtcaagccgggggggcgctggcgcgaggccgcctggcaactctgcgactactacctgccc
tacgcgctgggcggcggctacgtgctctcggccgacctggtgcactacctgcgcctcagccgcgactacctgcgcgcctggcacagcgagg
acgtgtctctgggcgcctggctggcgccggtggacgtccagcgggagcacgacccgcgcttcgacaccgaataccggtcccgcggctgcag
caaccagtacctggtgacgcacaagcagagcctggaggacatgctggagaagcacgcgacgctggcgcgcgagggccgcctgtgcaagc
gcgaggtgcagctgcgcctgtcctacgtgtacgactggtccgcgccgccctcgcagtgctgccagagaagggagggcatcccctga

The DNA sequence of c.510_517del:p.L170fs*268 *B3GALT6*

atgaagctgctgcggcgggcgtggcggcggcgggcggcgctaggcctgggcacgctggcgctgtgcggggcggcgctgctctacctggcg
cgctgcgcggccgagcccggggaccccagggcgatgtcgggccgcagcccgcctcccccgcgcccgcgcgcgccgccgccttcctggca
gtgctggtggccagcgcgccccgcgccgccgagcgccgcagcgtgatccgcagcacgtggcttgcgcggcgcggggccccgggcgacgtg
tgggcgcgctttgccgtgggcacggccggcctgggcgccgaggagcggcgcgccctggagcgggagcaggcgcggcacggggacctgct
gctgctgcccgcgctgcgcgacgcctacgaaaacctcacggccaaggtgctggccatgctggcctggctggacgagcacgtggccttcgag
ttcgtgctcaaggcggacgacgactccttcgcgcggctggacgcgctgctggccgagct**~~gcgcgccc~~**gcgagcccgcgcgccgccgccgcc
tctactggggcttcttctcgggccgcggccgcgtcaagccgggggggcgctggcgcgaggccgcctggcaactctgcgactactacctgccc
tacgcgctgggcggcggctacgtgctctcggccgacctggtgcactacctgcgcctcagccgcgactacctgcgcgcctggcacagcgagg
acgtgtctctgggcgcctggctggcgccggtggacgtccagcgggagcacgacccgcgcttcgacaccgaataccggtcccgcggctgcag
caaccagtacctggtgacgcacaagcagagcctggaggacatgctggagaagcacgcgacgctggcgcgcgagggccgcctgtgcaagc
gcgaggtgcagctgcgcctgtcctacgtgtacgactggtccgcgccgccctcgcagtgctgccagagaagggagggcatcccctgagccgc
cgcggcccggccctccgggacacctgcttcacccggcggcgccttggggcaggtgccgagcgggcgcactacgcccgggccccaaggccc
ccgtcccgcagccacgcttgtggtcgctgcgtcccggtctgcgtttgggagacccctgggggttgccggggcagcgcgccgtgtccaggtgg
aggtgcccgttcctggacctcagcgagcctgagccgggcccggccgcacgctgacccccgtgctgtccccgaccggctcacggggctgggc
tccgatcttccgtgtctcttatcagtggcgtttctcacgtctgcgtctcagatctaa

The amino acids sequence of wild-type β3Galt6 (NM_080605.4)

MKLLRRAWRRRAALGLGTLALCGAALLYLARCAAEPGDPRAMSGRSPPPPAPARAAAFLAVLVASAPRAAERR
SVIRSTWLARRGAPGDVWARFAVGTAGLGAEERRALEREQARHGDLLLLPALRDAYENLTAKVLAMLAWLDEH
VAFEFVLKADDDSFARLDALLAELRAREPARRRRLYWGFFSGRGRVKPGGRWREAAWQLCDYYLPYALGGGYV
LSADLVHYLRLSRDYLRAWHSEDVSLGAWLAPVDVQREHDPRFDTEYRSRGCSNQYLVTHKQSLEDMLEKHAT
LAREGRLCKREVQLRLSY VYDWSAPPSQ CCQRREGIP

The amino acids sequence of c.510_517del:p.L170fs*268-β3Galt6

MKLLRRAWRRRAALGLGTLALCGAALLYLARCAAEPGDPRAMSGRSPPPPAPARAAAFLAVLVASAPRAAERR
SVIRSTWLARRGAPGDVWARFAVGTAGLGAEERRALEREQARHGDLLLLPALRDAYENLTAKVLAMLAWLDEH
VAFEFVLKADDDSFARLDALLAELRARAPPPPLLGLLLGPRPRQAGGALARGRLATLRLLPALRAGRRLRALGRP
GALPAPQPRLPARLAQRGRVSGRLAGAGGRPAGARPALRHRIPVPRLQQPVPGDAQAEPGGHAGEARDAGA
RGPPVQARGAAAPVLRVRLVRAALAVLPEKGGHPLSRRGPALRDTCFTRRRLGAGAERAHYARAPRPPSRSHA
CGRCVPVCVWETPGGCRGSAPCPGGGARSWTSASLSRARPHADPRAVPDRLTGLGSDLPCLLSVA FLTSASQI

Supplementary Table 1   In silico missense prediction

algorithm	score	prediction
SIFT	0.034	Damaging
Polyphen-2_HDIV	0.989	Probably_damaging
Polyphen-2_HVAR	0.613	Possibly_damaging
LRT	0.081	Unknown
MutationTaster	1	Polymorphism
MutationAssessor	1.245	Low
FATHMM	0.86	Tolerable
PROVEAN	-3.59	Damaging
VEST3	0.24	Tolerable
MetaSVM	-1.038	Tolerable
MetaLR	0.101	Tolerable
M-CAP	0.394	Damaging
CADD	27	Damaging
DANN	0.998	Damaging
FATHMM_MKL	0.488	Tolerable
Eigen	-0.216	Tolerable
GenoCanyon	1	Damaging
fitCons	0.726	Damaging
GERP++	2.38	Conserved
phyloP	3.184	Conserved
phastCons	0.035	Nonconserved
SiPhy	7.545	Nonconserved
REVEL	0.117	Tolerable
ReVe	0.322	Tolerable
ClinPred	0.47779065	Benign
